# Identification of aldolase A as a potential diagnostic biomarker for colorectal cancer based on proteomic analysis using formalin-fixed paraffin-embedded tissue

**DOI:** 10.1007/s13277-016-5275-8

**Published:** 2016-07-28

**Authors:** Tetsushi Yamamoto, Mitsuhiro Kudo, Wei-Xia Peng, Hideyuki Takata, Hideki Takakura, Kiyoshi Teduka, Takenori Fujii, Kuniko Mitamura, Atsushi Taga, Eiji Uchida, Zenya Naito

**Affiliations:** 1 Pathological and Biomolecule Analyses Laboratory, Faculty of Pharmacy, Kindai University, Osaka, Japan; 2 Department of Integrated Diagnostic Pathology, Nippon Medical School, 1-1-5 Sendagi, Bunkyo-ku, Tokyo, 113-8602 Japan; 3 Departments of Gastrointestinal Hepato Biliary Pancreatic Surgery, Nippon Medical School, Tokyo, Japan

**Keywords:** Colorectal cancer, Shotgun proteomics, Formalin-fixed paraffin-embedded tissue, Aldolase A

## Abstract

Colorectal cancer (CRC) is one of the most common cancers worldwide, and many patients are already at an advanced stage when they are diagnosed. Therefore, novel biomarkers for early detection of colorectal cancer are required. In this study, we performed a global shotgun proteomic analysis using formalin-fixed and paraffin-embedded (FFPE) CRC tissue. We identified 84 candidate proteins whose expression levels were differentially expressed in cancer and non-cancer regions. A label-free semiquantitative method based on spectral counting and gene ontology (GO) analysis led to a total of 21 candidate proteins that could potentially be detected in blood. Validation studies revealed cyclophilin A, annexin A2, and aldolase A mRNA and protein expression levels were significantly higher in cancer regions than in non-cancer regions. Moreover, an in vitro study showed that secretion of aldolase A into the culture medium was clearly suppressed in CRC cells compared to normal colon epithelium. These findings suggest that decreased aldolase A in blood may be a novel biomarker for the early detection of CRC.

## Introduction

Colorectal cancer (CRC) is one of the most common cancers worldwide. If the tumor is limited to the mucosa or submucosa, CRC can be completely cured by endoscopic or surgical therapy; however, many patients are already at an advanced stage when they are diagnosed. Therefore, an early detection method is needed. Diagnostic blood tests based on detection of carcinoembryonic antigen (CEA) are now widely used for CRC, but the sensitivity of this biomarker in early-stage cancer is only 5–10 % [1, 2]. The identification of novel candidate molecules that are secreted by cancer cells may lead to the development of early detection methods and improved prognosis.

Formalin-fixed and paraffin-embedded (FFPE) tissues are archived in hospitals, together with detailed clinical information such as disease history, clinical examination results, and drug responses. FFPE tissues are routinely used in the diagnosis and research of diseases, including cancers, by conventional staining, immunohistochemistry (IHC), and in situ hybridization [3–5]. Previously, FFPE tissues were considered unsuitable for proteomic analysis. However, methods to isolate protein from FFPE tissues for proteomic analysis have been developed, and various FFPE tissues have been used in proteomic studies [6–17]. Thus, archived FFPE tissues may be useful for identifying new biomarkers.

In this study, we performed shotgun liquid chromatography (LC)/mass spectrometry (MS)-based global proteomic analysis using proteins from FFPE CRC tissue to identify proteins whose expression levels are modified in cancer regions. We identified aldolase A as a candidate protein with potential as a novel biomarker for early detection of CRC.

## Materials and methods

### Materials

The following materials were purchased from Wako Pure Chemical Industries (Osaka, Japan): guanidine hydrochloride, DTT, Tris, tris (2-carboxyethyl) phosphine hydrochloride (TCEP), and iodoacetamide (IAA). All other chemicals and reagents were purchased from Sigma Chemical Corp. (St. Louis, MO, USA).

### Patients and tissue specimens

The tissues used in this study were from 45 patients who underwent surgical resection for CRC at Nippon Medical School Hospital between October 2007 and August 2014. None of the patients received chemotherapy or radiation therapy prior to surgery and none had inflammatory colorectal disease such as colitis or infectious diseases. The pathological diagnosis and clinicopathological stage were determined according to the criteria of the World Health Organization [18]. After a histopathological analysis, 10 of these 45 cases were selected for proteomic analysis. Immunohistochemical analyses were performed on all 45 cases. Paraffin-embedded specimens were prepared for proteomic and immunohistochemical analyses. This study was carried out in accordance with the principles embodied in the Declaration of Helsinki, 2013, and the Japanese Society of Pathology Ethics Committee. Informed consent for the use of colorectal tissues was obtained from all patients.

### Protein extraction from FFPE tissue

**Fig. 1:**
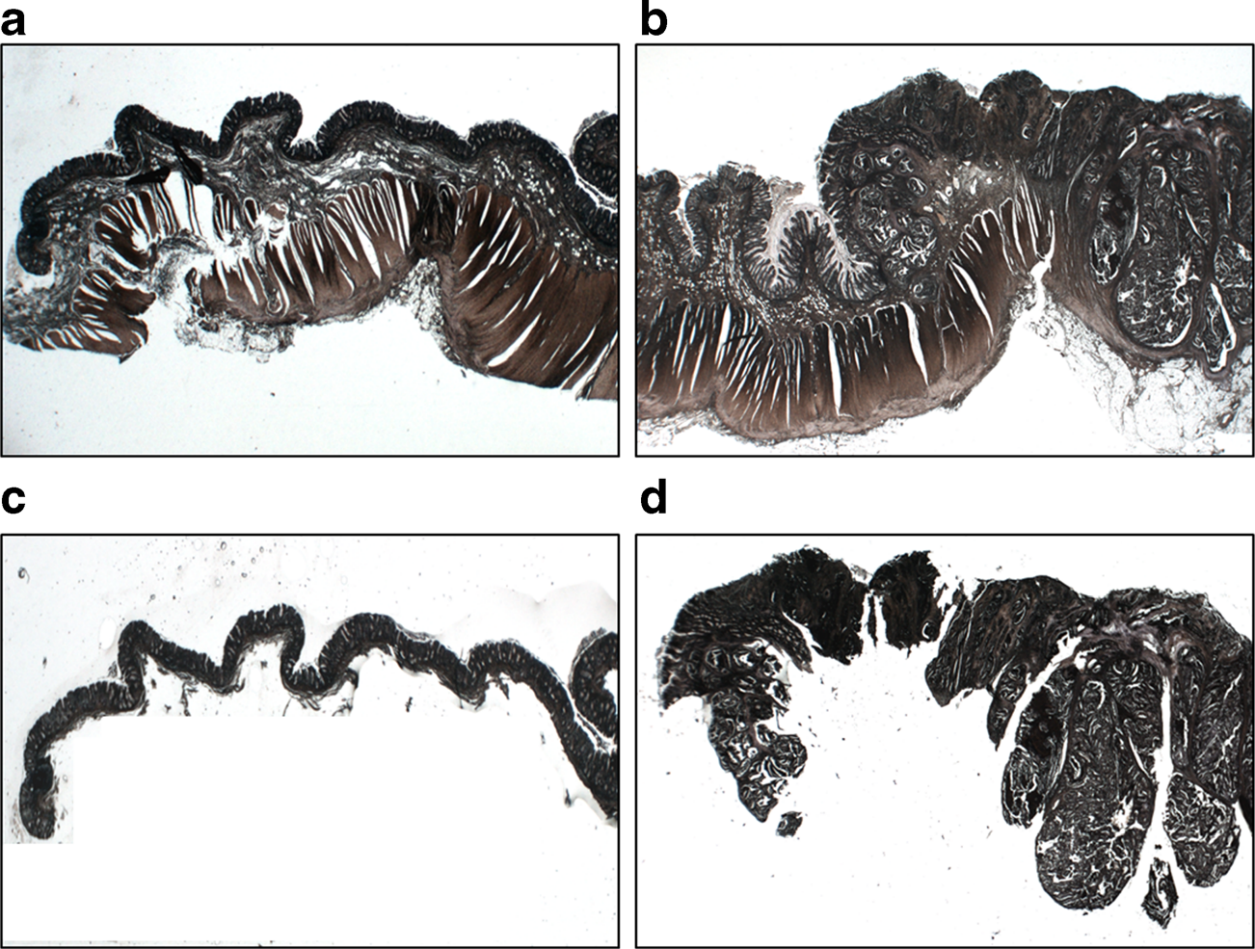
Manual dissection of FFPE colon tissue. Representative image shows non-cancer and cancer regions before (**a** and **b**, respectively) and after (**c** and **d**, respectively) dissection. Sections were stained with hematoxylin

FFPE CRC tissues from ten patients were used for proteomic analysis. Pathological and clinical information are shown in Table 1. Following histological examination of hematoxylin and eosin (H&E) sections, we separated the cancer regions from the non-cancer regions, which maintained normal structures. Sections (10 μm) were deparaffinized in xylene and rehydrated through a series of graded alcohols (100, 90, 80, and 70 %). After staining with Mayer’s hematoxylin for 5 min, cancer and non-cancer regions were manually dissected under a microscope (Fig. 1). Proteins were extracted from both cancer and non-cancer regions using lysis buffer (6 M guanidine-HCl, 40 mM Tris-HCl pH 8.2, and 65 mM DTT) according to a previous report [17]. Protein concentration was measured by the Bradford method.

**Table 1:** Clinical and pathological data of patients with CRC that contributed tissues for proteomic analysis

Patient No.	Age (year)	Gender	pTNM	pStaging	Tumor location	Number of identified proteins	
						Cancer region	Non-cancer region
1	63	M	T3N0M0	Stage IIA	S	49	26
2	53	M	T3N0M0	Stage IIA	S	38	38
3	77	F	T3N0M0	Stage IIA	S	50	34
4	53	M	T3N0M0	Stage IIA	S	69	52
5	76	F	T3N0M0	Stage IIA	S	52	22
6	51	M	T3N0M0	Stage IIA	A	22	4
7	67	F	TisN0M0	Stage 0	A	44	28
8	52	M	T3N0M0	Stage IIA	Ra	52	50
9	69	M	T3N0M0	Stage IIA	T	65	16
10	69	F	T3N0M0	Stage IIA	S	19	35

*M* male, *F* female, *A* ascending colon, *T* transverse colon, *S* sigmoid colon, *Ra* upper rectum

### In-solution trypsin digestion

A gel-free digestion was performed according to the protocol described by Bluemlein et al. [19], with slight modifications. Briefly, 10 μg of protein was extracted from each sample, reduced with 45 mM DTT and 20 mM TCEP, and then alkylated using 100 mM IAA. After alkylation, samples were digested with proteomics-grade trypsin (Agilent Technologies Inc., Santa Clara, CA, USA) at 37 °C for 24 h. Digests were purified using PepClean C-18 Spin Columns (Thermo, Rockford, IL, USA) according to the manufacturer’s protocol.

### LC-MS/MS analysis for protein identification

Approximately 2 μg of purified peptide samples were injected onto a peptide L-trap column (Chemicals Evaluation and Research Institute, Tokyo, Japan) using an HTS PAL autosampler (CTC Analytics, Zwingen, Switzerland) and further separated thorough an Advance-nano UHPLC (AMR Inc., Tokyo, Japan) using a reverse-phase C18-column (Zaplous column α, 3-μm diameter gel particles and 100 Å pore size, 0.1 × 150 mm; AMR). The mobile phase consisted of solution A (0.1 % formic acid in water) and solution B (acetonitrile). The flow rate was 500 nL/min, with a concentration gradient of acetonitrile from 5 % B to 35 % B over 120 min. Gradient-eluted peptides were analyzed using an amaZon ETD ion-trap mass spectrometer (Bruker Daltonics, Billerica, MA, USA). Data were acquired in a data-dependent manner, in which MS/MS fragmentation was performed on the ten most intense peaks of every full MS scan.

All MS/MS spectra data were searched against the SwissProt *Homo sapiens* database with Mascot (version_2.3.01; Matrix Science, London, UK). Search criteria were as follows: enzyme, trypsin; allowance of up to two missed cleavage peptides; mass tolerance ±0.5 Da and MS/MS tolerance ±0.5 Da; and modifications of cysteine carbamidomethylation, methionine oxidation, and N-formylation including formyl (K), formyl (R), and formyl (N terminus).

### Spectral counting analysis of identified proteins

To compare protein expression across all tissue samples, we used the spectral counting method. In this analysis, when not specific spectral peak could be identified, the expression level of that protein was taken as zero, as described in a previous report [20]. Fold-changes of expressed proteins in the base 2 logarithmic scale were calculated with Rsc based upon spectral counting [21]. Relative amounts of identified proteins were also calculated with the normalized spectral abundance factor (NSAF) [22]. Candidate proteins with modified expression levels in the cancer region were chosen to ensure that the Rsc satisfied >1 or <−1, which corresponded to fold changes of >2 or <0.5.

### Bioinformatics

Functional annotations for the identified proteins with modified expression levels in the cancer region were processed using the Database for annotation, visualization, and integrated discovery (DAVID) version 6.7 (http://david.abcc.ncifcrf.gov/home.jsp) [23–25].

### Quantitative RT-PCR

Total RNA was extracted from FFPE CRC tissue with the RNeasy FFPE Kit (QIAGEN, Valencia, CA, USA) and from CRC cell lines with the GenElute Mammalian Total RNA Miniprep Kit (Sigma). cDNA was synthesized using the SuperScript VILO cDNA Synthesis Kit for FFPE tissue and the High Capacity cDNA Reverse Transcription kit for the CRC cell line according to the manufacturer’s protocols (Life Technologies Japan, Tokyo, Japan). To measure expression of aldolase A, cyclophilin A, and annexin A2 mRNA, quantitative reverse transcription PCR (qRT-PCR) was performed with the 7500 system (Applied Biosystems, Foster City, CA, USA). Primers and TaqMan probes for aldolase A (Hs00765620_m1), cyclophilin A (Hs04194521_s1), annexin A2 (Hs00743063_s1), and 18S rRNA (Hs03928990_g1) were used with the TaqMan Gene Expression Assay. qRT-PCR results were expressed relative to an internal standard concentration as a ratio of target/18S rRNA. Gene expression was measured in triplicate.

### Immunohistochemistry

FFPE CRC tissues from 46 patients were used for a validation analysis. Pathological and clinical information are shown in Table 2. Paraffin-embedded tissue sections (3 μm) were subjected to immunostaining using a Histofine Simple Stain MAX-PO (R) kit (Nichirei, Tokyo, Japan) for identifying aldolase A, annexin A2, and cyclophilin A. After deparaffinization, sections were pretreated in an autoclave at 121 °C for 15 min in 10 mM citrate buffer (pH 6.0) for cyclophilin A. Endogenous peroxidase activity was blocked by incubation for 30 min with 0.3 % hydrogen peroxide in methanol. Tissue sections were then incubated with the anti-ALDOA antibody (1:150 dilution; Atlas Antibodies, Stockholm, Sweden) for aldolase A, anti-cyclophilin A antibody (1:150 dilution; Novus Biologicals, Littleton, CO, USA), or anti-annexin A2 antibody (1:400 dilution; Cell Signaling Technology, Inc., Danvers, MA, USA) in phosphate-buffered saline containing 1 % bovine serum albumin for 16 h at 4 °C. Bound antibodies were detected with the Simple Stain MAX-PO (R) with diaminobenzidine tetrahydrochloride as the substrate. Sections were then counterstained with Mayer’s hematoxylin. Two investigators (TY and HT) evaluated all the sections separately in a blinded manner. Sections were scored for both intensity (0, no stain; 1, weak; 2, moderate; and 3, strong) and percentage of epithelial cells that stained positive (0, 0–5 %; 1, 6–25 %; 2, 26–50 %; 3, 51–75 %; and 4, 76–100 %). Scores were derived from the sum of the intensity and percentage of immunoreactive cells [15].

**Table 2:** Clinical and pathological data of patients with CRC that contributed tissues for immunohistochemical analysis

Patient No.	Age(year)	Gender	pTNM	pStaging	Tumor location
1	67	F	TisN0M0	Stage 0	A
2	66	F	TisN0M0	Stage 0	D
3	61	F	TisN0M0	Stage 0	S
4	70	M	TisN0M0	Stage 0	T
5	64	F	TisN0M0	Stage 0	T
6	63	M	T2N0M0	Stage I	Rb
7	58	F	T2N0M0	Stage I	Rb
8	45	F	T2N0M0	Stage I	Rb
9	67	F	T2N0M0	Stage I	Ra
10	87	F	T2N0M0	Stage I	Ra
11	67	F	T2N0M0	Stage I	S
12	70	M	T1N0M0	Stage I	S
13	75	F	T1N0M0	Stage I	T
14	82	M	T1N0M0	Stage I	T
15	63	M	T3N0M0	Stage IIA	S
16	53	M	T3N0M0	Stage IIA	S
17	77	F	T3N0M0	Stage IIA	S
18	53	M	T3N0M0	Stage IIA	S
19	76	F	T3N0M0	Stage IIA	S
20	51	M	T3N0M0	Stage IIA	A
21	52	M	T3N0M0	Stage IIA	Ra
22	69	M	T3N0M0	Stage IIA	T
23	69	F	T3N0M0	Stage IIA	S
24	92	M	T3N0M0	Stage IIA	T
25	64	M	T2N1aM0	Stage IIIA	Ra
26	73	F	T2N1bM0	Stage IIIA	Ra
27	64	M	T3N1aM0	Stage IIIB	Ra
28	50	F	T3N1bM0	Stage IIIB	S
29	51	M	T3N1bM0	Stage IIIB	A
30	64	M	T3N1aM0	Stage IIIB	S
31	64	F	T3N1bM0	Stage IIIB	Ra
32	78	F	T3N1aM0	Stage IIIB	A
33	72	F	T4aN1AM0	Stage IIIB	C
34	52	M	T3N1bM0	Stage IIIB	Ra
35	85	F	T4bN1aM0	Stage IIIC	A
36	70	M	T4aN2aM0	Stage IIIC	S
37	81	F	T4aN0M1a	Stage IVA	T
38	71	F	T4aN2aM1a	Stage IVA	A
39	49	M	T3N2aM1a	Stage IVA	C
40	78	M	T3N1bM1a	Stage IVA	Ra
41	77	M	T4aN2aM1a	Stage IVA	T
42	57	M	T3N2bM1b	Stage IVB	Ra
43	72	F	T4aN0M1b	Stage IVB	D
44	71	F	T4bN2bM1b	Stage IVB	C
45	70	M	T3N0M1b	Stage IVB	S

*M* male, *F* female, *A* ascending colon, *T* transverse colon, *S* sigmoid colon, *Ra* upper rectum, *Rb* lower rectum, *D* descending colon, *C* cecum

### CRC cell lines

CRC cell lines DLD-1, SW480, and SW620 and normal human colon epithelial cell line CCD 841 CoN were purchased from the American Type Culture Collection (Manassas, VA, USA). All cells were cultured in RPMI 1640 medium supplemented with 10 % fetal bovine serum (FBS) (Gibco, Carlsbad, CA, USA) in an atmosphere containing 5 % CO_2_.

### Protein preparation

CRC cells were plated at a density of 5 × 10^5^ cells per dish in a 100-mm dish and grown in culture medium. After 72 h, cells were solubilized in urea lysis buffer (7 M urea, 2 M thiourea, 5 % CHAPS, and 1 % Triton X-100). To quantify secreted protein, the culture medium of CRC cells was collected after 72 h.

### Western blot analysis

The cell extract, culture medium, and commercial human normal serum (ImmunoBioScience Corp., Mukilteo, WA) were subjected to SDS-PAGE under reducing conditions. The separated proteins were transferred to polyvinylidene fluoride transfer membranes. Membranes were incubated with an anti-annexin A2 rabbit monoclonal antibody, anti-aldolase A rabbit monoclonal antibody, or anti-cyclophilin A antibody (Cell Signaling Technology Inc., Beverly, MA, USA) at 4 °C overnight. Membranes were then washed and incubated with HRP-conjugated anti-rabbit IgG antibody (American Qualex, San Clemente, CA). After washing, blots were visualized by enhanced chemiluminescence and detected using a myECL Imager system (ThermoFisher Scientific). The same membranes were reprobed with anti-β-actin antibody (Sigma) to confirm equal loading of the proteins. All Western blot analyses were performed three times.

### Statistical analysis

All data are presented as the mean ± standard error of the mean. Data between two groups were compared with the unpaired *t* test. Values of *P < 0.05 and **P < 0.001 were considered significant in all analyses. Computations were performed with GraphPad Prism version 5 (GraphPad Software, La Jolla, CA, USA).

## Results

### Protein identification and profiles in cancer and non-cancer regions of CRC tissue

**Fig. 2:**
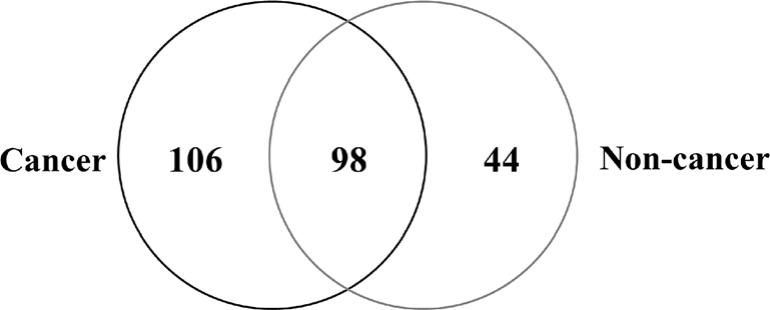
Venn map of proteins identified from FFPE colorectal cancer tissue. We identified 204 proteins in the cancer regions and 142 in the non-cancer regions

To investigate the molecular profile of proteins expressed in relation to cancer progression, we performed shotgun proteomic analysis using FFPE CRC tissue. We used manual dissection to separate the cancer regions and non-cancer regions from FFPE CRC tissues and examined the protein expression not only in cells but also in the stromal tissue surrounding cells (Fig. 1). We successfully identified proteins in both cancer and non-cancer regions of FFPE tissues (Table 1). Figure 2 shows the Venn map for the proteins identified.

### Semiquantitative comparison of proteins identified in cancer and non-cancer regions of CRC tissue

**Fig. 3:**
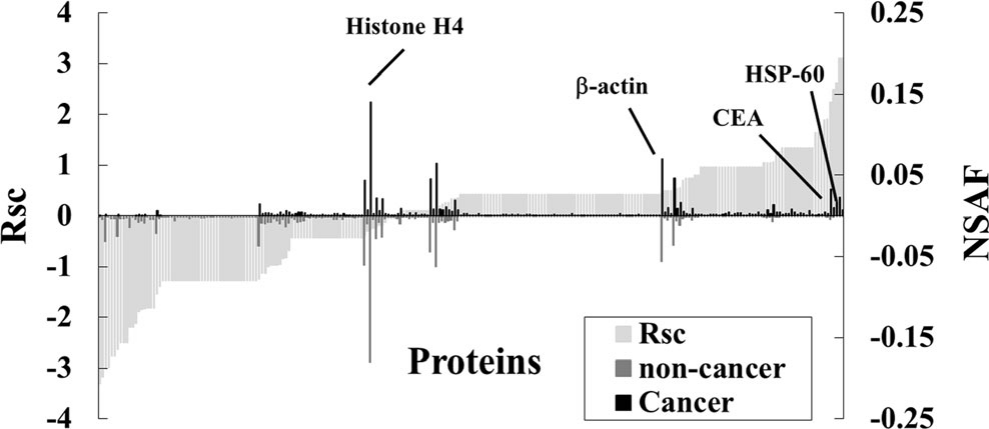
Semiquantitative comparison of proteins identified in FFPE colon tissue. Rsc and normalized spectral abundance factor (NSAF) values calculated for the proteins identified (*x*-axis). Comparison of protein expression in cancer versus non-cancer tissue. Proteins highly expressed in either cancer or non-cancer regions were plotted near the right or left side of the *x*-axis

We next performed a label-free semiquantitative method based on spectral counting to identify proteins with modified expression levels in the cancer region. The Rsc value was plotted against the corresponding protein (*x*-axis) in increasing order from left to right for proteins identified in the cancer and non-cancer regions. Positive and negative Rsc values indicate increased and decreased expression, respectively, in the cancer region. In Fig. 3, the NSAF value (bar) was plotted against the corresponding protein (*x*-axis) and the NSAF values of the cancer regions (black bar) and non-cancer regions (gray bar). Proteins are indicated above and below the *x*-axis. Proteins with either a high positive or negative Rsc value were considered as potential early detection markers for CRC.

A total of 84 differentially expressed proteins were identified in the cancer regions (Table 3). Representative proteins that were upregulated included known tumor markers such as 60 kDa heat shock protein (HSP60) and carcinoembryonic antigen-related cell adhesion molecule 5 (CEAM5, also known as CEA). By contrast, expression levels of housekeeping proteins such as β-actin and histone H4 did not change.

**Table 3:** Differentially expressed proteins in cancer region of CRC samples

No.	ID	Accession number and description	No. of amino acids	Spectral counting		
				Non-cancer	Cancer	Fold change (Rsc)
1	FCGBP_HUMAN	(Q9Y6R7) IgGFc-binding protein	5405	15	1	−3.307086
2	K1C10_HUMAN	(P13645) Keratin, type I cytoskeletal 10	584	7	0	−3.163409
3	H3C_HUMAN	(Q6NXT2) Histone H3.3C	135	12	1	−3.006964
4	CO1A1_HUMAN	(P02452) Collagen alpha-1(I) chain	1464	6	0	−2.975121
5	K22E_HUMAN	(P35908) Keratin, type II cytoskeletal 2 epidermal	639	5	0	−2.759125
6	K2C6B_HUMAN	(P04259) Keratin, type II cytoskeletal 6B	564	5	0	−2.759125
7	H2B1A_HUMAN	(Q96A08) Histone H2B type 1-A	127	9	1	−2.630943
8	K1C14_HUMAN	(P02533) Keratin, type I cytoskeletal 14	472	4	0	−2.505717
9	K1C17_HUMAN	(Q04695) Keratin, type I cytoskeletal 17	432	4	0	−2.505717
10	K2C5_HUMAN	(P13647) Keratin, type II cytoskeletal 5	590	4	0	−2.505717
11	CRIP1_HUMAN	(P50238) Cysteine-rich protein 1	77	3	0	−2.198995
12	MUC2_HUMAN	(Q02817) Mucin-2	5179	3	0	−2.198995
13	ATPA_HUMAN	(P25705) ATP synthase subunit alpha, mitochondrial	553	6	1	−2.125742
14	K2C1B_HUMAN	(Q7Z794) Keratin, type II cytoskeletal 1b	578	13	4	−1.887275
15	LMNA_HUMAN	(P02545) Prelamin-A/C	664	10	3	−1.84682
16	K1C9_HUMAN	(P35527) Keratin, type I cytoskeletal 9	623	15	5	−1.827612
17	ACTN4_HUMAN	(O43707) Alpha-actinin-4	911	2	0	−1.810107
18	GLUC_HUMAN	(P01275) Glucagon	180	2	0	−1.810107
19	RS7_HUMAN	(P62081) 40S ribosomal protein S7	194	2	0	−1.810107
20	K2C1_HUMAN	(P04264) Keratin, type II cytoskeletal 1	644	39	18	−1.541287
21	CLCA1_HUMAN	(A8K7I4) Calcium-activated chloride channel regulator 1	914	7	3	−1.393723
22	CAH1_HUMAN	(P00915) Carbonic anhydrase 1	261	1	0	−1.277731
23	GSLG1_HUMAN	(Q92896) Golgi apparatus protein 1	1179	1	0	−1.277731
24	LAMB3_HUMAN	(Q13751) Laminin subunit beta-3	1172	1	0	−1.277731
25	MK15_HUMAN	(Q8TD08) Mitogen-activated protein kinase 15	544	1	0	−1.277731
26	RS30_HUMAN	(P62861) 40S ribosomal protein S30	59	1	0	−1.277731
27	ZHANG_HUMAN	(Q9NS37) CREB/ATF bZIP transcription factor	354	1	0	−1.277731
28	ZN408_HUMAN	(Q9H9D4) Zinc finger protein 408	720	1	0	−1.277731
29	PGBM_HUMAN	(P98160) Basement membrane-specific heparan sulfate proteoglycan core protein	4391	1	0	−1.277731
30	DPYL2_HUMAN	(Q16555) Dihydropyrimidinase-related protein 2	572	1	0	−1.277731
31	RL31_HUMAN	(P62899) 60S ribosomal protein L31	125	1	0	−1.277731
32	HNRPL_HUMAN	(P14866) Heterogeneous nuclear ribonucleoprotein L	589	1	0	−1.277731
33	FLNA_HUMAN	(P21333) Filamin-A	2647	1	0	−1.277731
34	CO6A2_HUMAN	(P12110) Collagen alpha-2(VI) chain	1019	1	0	−1.277731
35	ARPC5_HUMAN	(O15511) Actin-related protein 2/3 complex subunit 5	151	1	0	−1.277731
36	RS23_HUMAN	(P62266) 40S ribosomal protein S23	143	1	0	−1.277731
37	MYH14_HUMAN	(Q7Z406) Myosin-14	1995	1	0	−1.277731
38	EF2_HUMAN	(P13639) Elongation factor 2	858	1	0	−1.277731
39	ECHB_HUMAN	(P55084) Trifunctional enzyme subunit beta, mitochondrial	474	1	0	−1.277731
40	FA48A_HUMAN	(Q8NEM7) Protein FAM48A	779	1	0	−1.277731
41	SETX_HUMAN	(Q7Z333) Probable helicase senataxin	2677	1	0	−1.277731
42	GFAP_HUMAN	(P14136) Glial fibrillary acidic protein	432	1	0	−1.277731
43	PERI_HUMAN	(P41219) Peripherin	470	1	0	−1.277731
44	POTEE_HUMAN	(Q6S8J3) POTE ankyrin domain family member E	1075	1	0	−1.277731
45	TTHY_HUMAN	(P02766) Transthyretin	147	1	0	−1.277731
46	CAH2_HUMAN	(P00918) Carbonic anhydrase 2	260	1	0	−1.277731
47	ENPL_HUMAN	(P14625) Endoplasmin	803	1	0	−1.277731
48	HS71L_HUMAN	(P34931) Heat shock 70 kDa protein 1-like	641	1	0	−1.277731
49	CBR1_HUMAN	(P16152) Carbonyl reductase [NADPH] 1	277	1	0	−1.277731
50	SPTA2_HUMAN	(Q13813) Spectrin alpha chain, brain	2472	1	0	−1.277731
51	HNRCL_HUMAN	(O60812) Heterogeneous nuclear ribonucleoprotein C-like 1	293	1	0	−1.277731
52	UGGG1_HUMAN	(Q9NYU2) UDP-glucose:glycoprotein glucosyltransferase 1	1555	1	0	−1.277731
53	OST48_HUMAN	(P39656) Dolichyl-diphosphooligosaccharide--protein glycosyltransferase 48 kDa subunit	456	1	0	−1.277731
54	ACTA_HUMAN	(P62736) Actin, aortic smooth muscle	377	40	23	−1.238478
55	AGR2_HUMAN	(O95994) Anterior gradient protein 2 homolog	175	4	2	−1.124439
56	TBB2C_HUMAN	(P68371) Tubulin beta-2C chain	445	12	7	−1.124173
57	KCRB_HUMAN	(P12277) Creatine kinase B-type	381	8	5	−1.001492
58	HSP7C_HUMAN	(P11142) Heat shock cognate 71 kDa protein	643	1	5	1.051122
59	PPIA_HUMAN	(P62937) Peptidyl-prolyl cis-trans isomerase A	165	1	5	1.051122
60	SBP1_HUMAN	(Q13228) Selenium-binding protein 1	472	1	5	1.051122
61	G3P_HUMAN	(P04406) Glyceraldehyde-3-phosphate dehydrogenase	335	6	19	1.0692919
62	ANXA2_HUMAN	(P07355) Annexin A2	339	1	6	1.2666356
63	MDHM_HUMAN	(P40926) Malate dehydrogenase, mitochondrial	338	1	6	1.2666356
64	ALDOA_HUMAN	(P04075) Fructose-bisphosphate aldolase A	364	0	3	1.3418115
65	LDHA_HUMAN	(P00338) L-lactate dehydrogenase A chain	332	0	3	1.3418115
66	RL7_HUMAN	(P18124) 60S ribosomal protein L7	248	0	3	1.3418115
67	S10A8_HUMAN	(P05109) Protein S100-A8	93	0	3	1.3418115
68	RL12_HUMAN	(P30050) 60S ribosomal protein L12	165	0	3	1.3418115
69	TBB1_HUMAN	(Q9H4B7) Tubulin beta-1 chain	451	0	3	1.3418115
70	TAGL2_HUMAN	(P37802) Transgelin-2	199	0	3	1.3418115
71	LDHA_HUMAN	(P00338) L-lactate dehydrogenase A chain	332	0	3	1.3418115
72	DHSA_HUMAN	(P31040) Succinate dehydrogenase [ubiquinone] flavoprotein subunit, mitochondrial	664	0	3	1.3418115
73	S10A9_HUMAN	(P06702) Protein S100-A9	114	0	3	1.3418115
74	TBA1A_HUMAN	(Q71U36) Tubulin alpha-1 A chain	451	0	3	1.3418115
75	FIBA_HUMAN	(P02671) Fibrinogen alpha chain	866	0	4	1.6480523
76	CEAM5_HUMAN	(P06731) Carcinoembryonic antigen-related cell adhesion molecule 5	702	0	4	1.6480523
77	RL1D1_HUMAN	(O76021) Ribosomal L1 domain-containing protein 1	490	0	4	1.6480523
78	1433B_HUMAN	(P31946) 14–3-3 protein beta/alpha	246	0	5	1.9009786
79	ACTN1_HUMAN	(P12814) Alpha-actinin-1	892	1	10	1.9060767
80	DEF1_HUMAN	(P59665) Neutrophil defensin 1	94	1	13	2.2513044
81	CH60_HUMAN	(P10809) 60 kDa heat shock protein, mitochondrial	573	2	23	2.5000084
82	H2B1C_HUMAN	(P62807) Histone H2B type 1-C/E/F/G/I	126	0	9	2.620238
83	H31_HUMAN	(P68431) Histone H3.1	136	0	13	3.1011611
84	TBB5_HUMAN	(P07437) Tubulin beta chain	444	0	13	3.1011611

Expression levels of these 84 proteins were more than two-fold higher or lower in cancer regions compared to non-cancer regions of CRC samples

### Functional annotation of differentially expressed proteins in cancer regions of CRC tissue

**Fig. 4:**
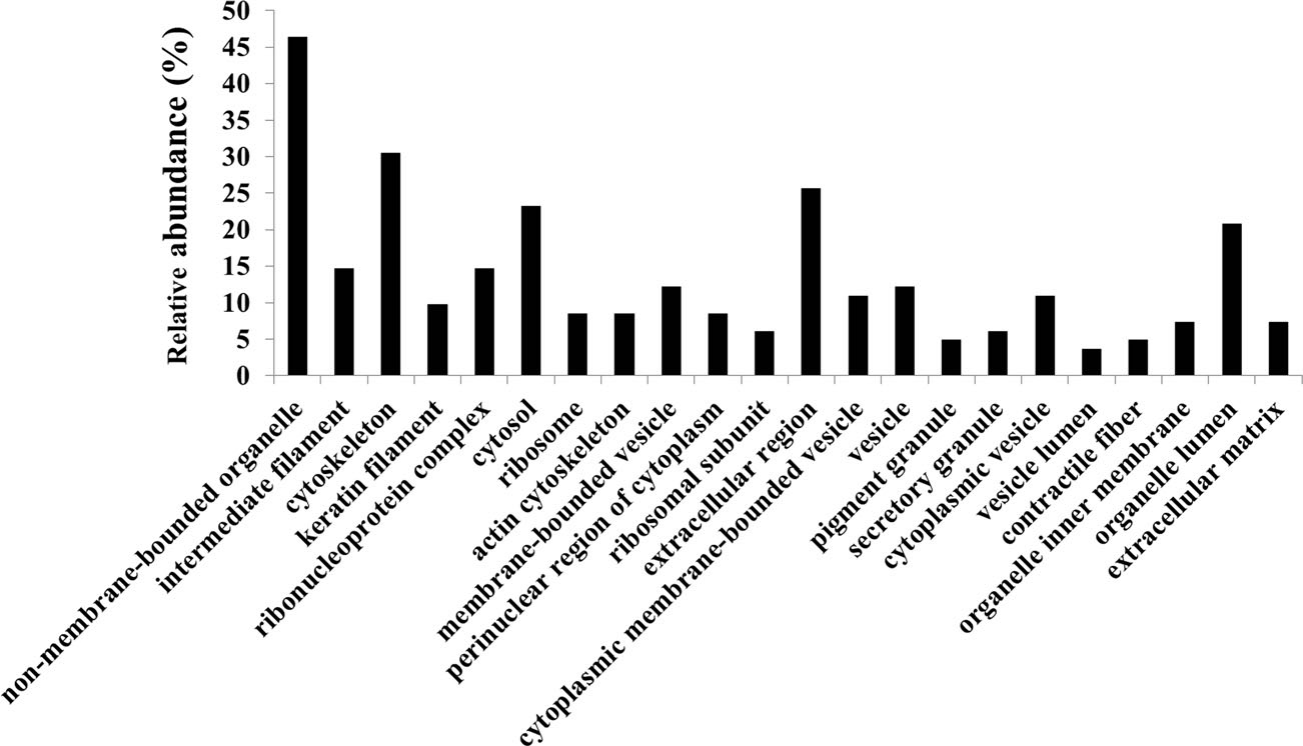
Analysis of GO cellular components of identified proteins. Protein assignments to GO cellular component categories are shown only for significant categories (*p* < 0.05)

DAVID was used to perform GO analyses for the differentially expressed proteins identified for each cellular component (Fig. 4). Functional annotations were counted by normalizing to the total number of identified proteins. Based on the cellular component classification, we focused on 21 proteins classified in the extracellular region, which could potentially be detected in the blood of patients with CRC (Table 4).

**Table 4:** Proteins categorized as extracellular region proteins

No.	Accession number and description	Fold change (Rsc)
1	(Q9Y6R7) IgGFc-binding protein	−3.3070855
2	(P02452) Collagen alpha-1(I) chain	−2.9751214
3	(Q02817) Mucin-2	−2.1989951
4	(O43707) Alpha-actinin-4	−1.8101074
5	(P01275) Glucagon	−1.8101074
6	(A8K7I4) Calcium-activated chloride channel regulator 1	−1.3937233
7	(Q13751) Laminin subunit beta-3	−1.2777305
8	(Q8TD08) Mitogen-activated protein kinase 15	−1.2777305
9	(P98160) Basement membrane-specific heparan sulfate proteoglycan core protein	−1.2777305
10	(P21333) Filamin-A	−1.2777305
11	(P12110) Collagen alpha-2(VI) chain	−1.2777305
12	(P02766) Transthyretin	−1.2777305
13	(P00918) Carbonic anhydrase 2	−1.2777305
14	(O95994) Anterior gradient protein 2 homolog	−1.1244392
15	(P62937) Peptidyl-prolyl cis-trans isomerase A	1.05112197
16	(P07355) Annexin A2	1.26663565
17	(P04075) Fructose-bisphosphate aldolase A	1.34181152
18	(P02671) Fibrinogen alpha chain	1.64805231
19	(P12814) Alpha-actinin-1	1.90607666
20	(P59665) Neutrophil defensin 1	2.25130442
21	(P10809) 60 kDa heat shock protein, mitochondrial	2.50000843

Twenty-one proteins that were differentially expressed in cancer regions of CRC samples were classified as extracellular region proteins by gene ontology analysis

### qRT-PCR analysis of aldolase A, cyclophilin A, and annexin A2 in cancer and non-cancer regions of CRC tissue

**Fig. 5:**
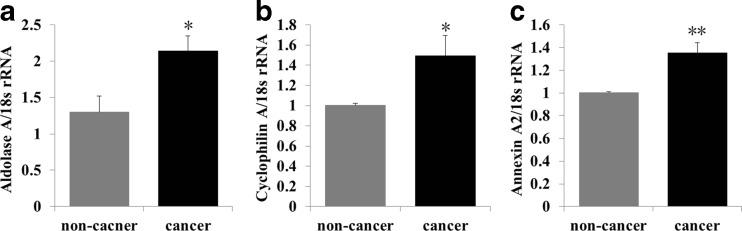
qPCR analysis of spectral counting results. Aldolase A, cyclophilin A, and annexin A2 were selected for confirmation. Expression levels of aldolase A, cyclophilin A, and annexin A2 mRNA were significantly higher in cancer compared to non-cancer regions (**a**–**c**). *p < 0.05, **p < 0.001

Fructose-bisphosphate aldolase A (aldolase A), peptidyl-prolyl cis-trans isomerase A (also known as cyclophilin A), and annexin A2 were upregulated in cancer regions (Table 3) and consequently selected as candidate diagnostic biomarkers. To confirm the higher expression levels of the candidate proteins in cancer regions compared to non-cancer regions, we performed qRT-PCR analysis. Expression levels of the mRNAs for these proteins were significantly higher in cancer compared to non-cancer regions (Fig. 5a–c).

### IHC analysis of aldolase A, cyclophilin A, and annexin A2 in cancer and non-cancer regions of CRC tissue

**Fig. 6:**
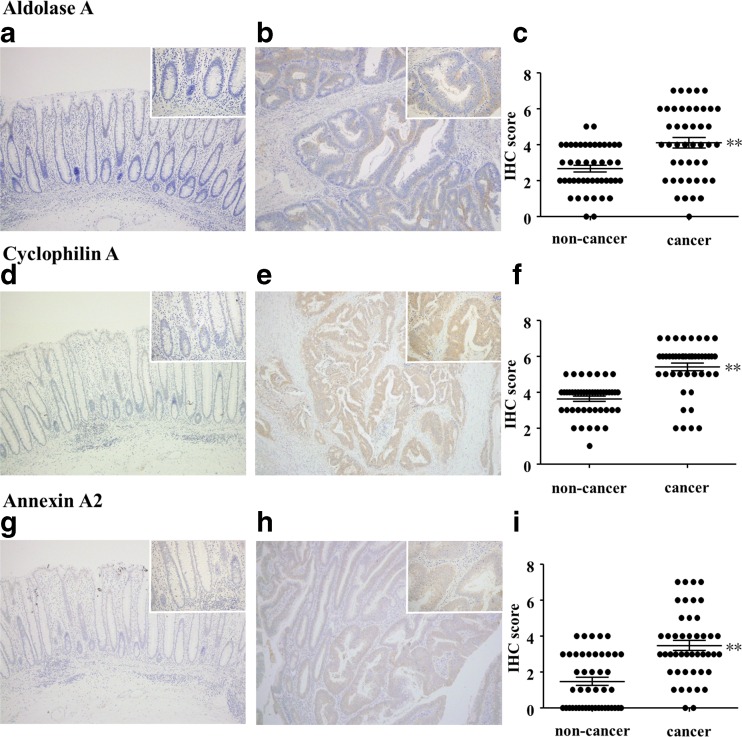
IHC validation of spectral counting results. Expression levels of aldolase A, cyclophilin A, and annexin A2 were validated by IHC. Representative images of non-cancer regions (**a**, **d**, and **g**) and cancer regions (**b**, **e**, and **h**). Graphs of IHC scores are shown in (**c**), (**f**), and (**i**). Cancer regions showed significantly stronger expression of these three proteins, which is consistent with spectral counting data. *p < 0.05; **p < 0.001. Original magnification, 100×; insets, 400×

We next performed IHC analysis of the three candidate proteins for validation with tissues from 45 CRC cases (Table 2). IHC analysis revealed that expression levels of all three proteins were significantly higher in cancer regions compared to non-cancer regions (Fig. 6).

### Expression of aldolase A, cyclophilin A, and annexin A2 in CRC cell lines

**Fig. 7:**
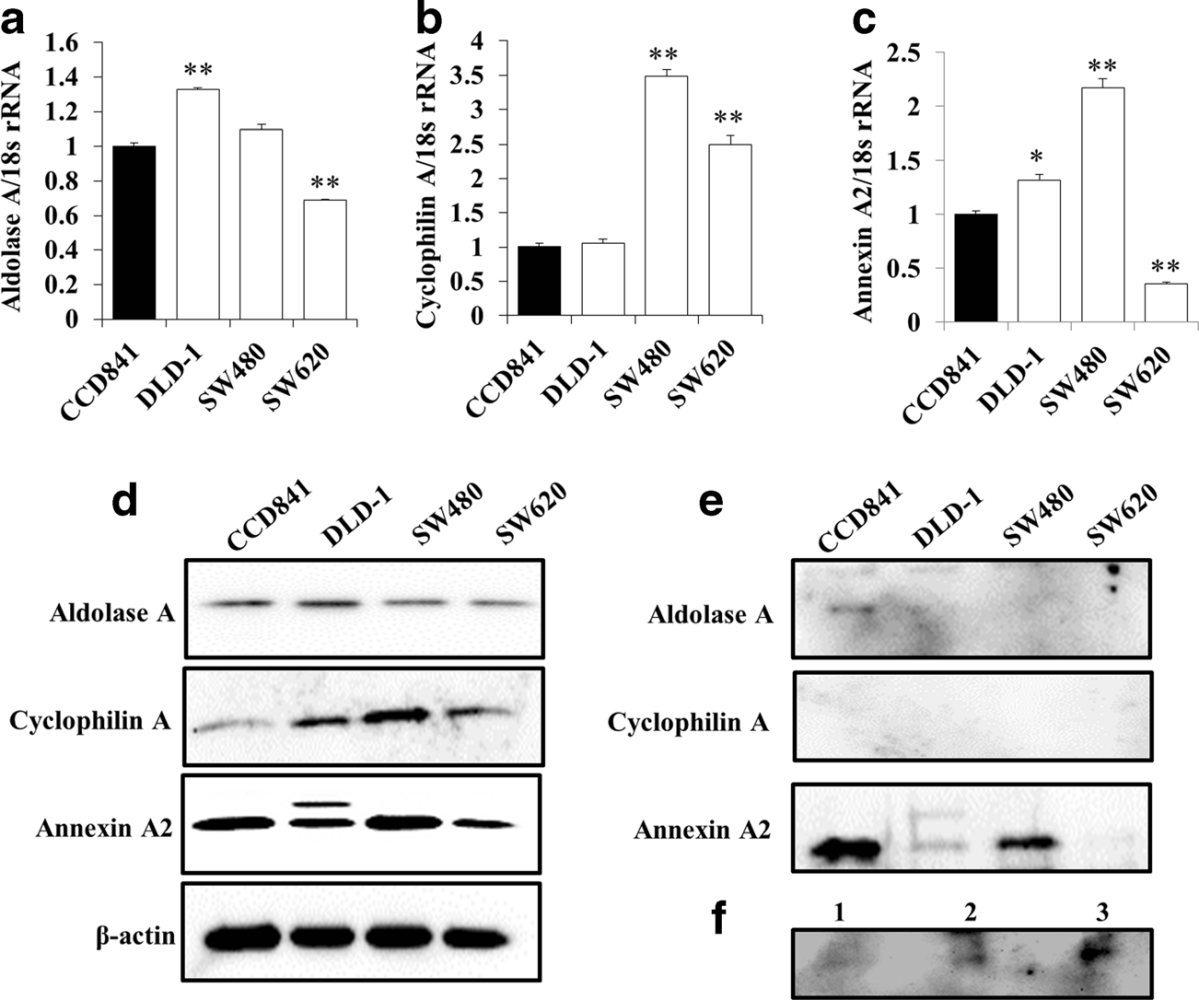
Expression of the three candidate proteins in CRC cell lines. Expression levels of aldolase A, cyclophilin A, and annexin A2 were examined by qRT-PCR and western blot. Expression levels of aldolase A (**a**), cyclophilin A (**b**), and annexin A2 (**c**) mRNA in CRC cells were almost all significantly higher than in normal colon epithelium. *p < 0.05; **p < 0.001. Expression levels of the three candidate proteins in cell extracts from CRC cell lines were similar to mRNA expression levels (**d**). Aldolase A was only detected in the culture medium of normal colon epithelium (**e**, upper panel). Cyclophilin A was not detected in any of the examined culture medium samples (**e**, middle panel). The level of annexin A2 in the culture medium appeared to depend on the level in the cell extract for each cell line (**e**, lower panel). Aldolase A was detected in human normal serum, and the level of aldolase A detected depended on the volume of serum loaded (**f**, lane 1, 10 μg serum; lane 2, 20 μg serum; lane 3, 30 μg serum)

To investigate whether the candidate proteins were suitable as biomarkers, their mRNA and protein expression levels in CRC cell lines were determined by qRT-PCR analysis and western blotting. All tested cell lines expressed the three candidate proteins, and their expression levels in CRC cells tended to be higher than in normal colon epithelial cells (Fig. 7a–d). Aldolase A was not detected in the culture medium of the three CRC cell lines, but it was detected in normal colon epithelial cells (Fig. 7e, upper panel). Cyclophilin A was not detected in the culture medium of any cell lines (Fig. 7e, middle panel). The level of annexin A2 in the culture medium appeared to depend on the level of annexin A2 in the cell extract for each cell line (Fig. 7e, lower panel). Finally, we detected aldolase A in normal human serum and confirmed that the level depended on the loaded serum volume (Fig. 7f, lane 1–3).

## Discussion

In this study, we used a gel-free LC-MS-based proteomics approach to identify potential biomarkers for cancer in FFPE CRC tissues. We used semiquantitative methods based on spectral counting to detect 84 proteins in which expression levels were altered >2-fold in cancer compared to non-cancer regions of CRC tissue (Table 3). CEA, a known tumor marker used in diagnostic blood tests for CRC, was among these proteins. Thus, the approach used in this study could potentially be used to identify novel biomarker candidates.

To identify early detection markers for CRC that can be detected by diagnostic blood tests, we focused on proteins in the “extracellular region” category of cellular components based on the results of GO analysis (Table 4). These proteins are secreted into the extracellular space and thus may potentially be detected in blood. These criteria led us to select aldolase A, cyclophilin A, and annexin A2 as candidates. We did not include HSP60, neutrophil defensin1, or alpha-actinin-1, because previous reports had already suggested that these proteins might be biomarkers for CRC [26–29].

Validation studies revealed that mRNA and protein expression levels of the three candidate proteins were significantly higher in the cancer region compared to non-cancer regions (Figs. 5 and 6). Moreover, to evaluate whether these proteins were useful as biomarkers, we also investigated their mRNA and protein expression levels in CRC cell lines and secretion of the proteins into the medium. Cyclophilin A was not secreted into the culture medium of all tested CRC cell lines (Fig. 7e). Thus, cyclophilin A is not suitable as a diagnostic blood tumor marker. On the other hand, annexin A2 expression in SW480 cells derived from the primary tumor site of a CRC patient was higher than expression in SW620 cells that were established from a metastatic site in the same patient (Fig. 7c–e). Decreased annexin A2 expression in cancer cells could play a role in the progression and metastasis of CRC and may be useful for predicting metastasis.

Although mRNA and protein expression of aldolase A were detected in all tested cell lines (Fig. 7a and d), aldolase A protein was not detected in the culture medium of the three CRC cell lines, while it was clearly detected in the culture medium of normal colon epithelial cells (Fig. 7e). Moreover, aldolase A was detected in normal human serum (Fig. 7f). These results suggest that secretion of aldolase A may be suppressed by dysfunction of aldolase A due to accumulated genetic mutations during the carcinogenesis process of CRC. Therefore, decreased aldolase A levels in blood may be useful as a biomarker for the early diagnosis of CRC.

Aldolase A is a glycolytic enzyme and contributes to various cellular functions related to muscle maintenance, cell shape and mobility regulation, striated muscle contraction, actin filament organization, and ATP biosynthesis [30–40]. There are several reports of elevated expression of aldolase A in the serum of patients with malignant tumors, such as those with lung and renal cancer [41, 42]. However, the expression kinetics and functions of aldolase A in CRC are not well understood. To our knowledge, this is the first report of decreased secretion of aldolase A in CRC. Thus, further studies are necessary to clarify the decrease in aldolase A levels in the blood of CRC patients in order to determine if this protein can be used as an early detection biomarker for CRC.

## Conclusion

In conclusion, we identified 248 proteins from FFPE CRC tissues using global shotgun proteomics. A label-free semiquantitative method based on spectral counting and GO identified 21 candidate early detection biomarkers for CRC that could potentially be detected in blood. Validation studies revealed that cyclophilin A, annexin A2, and aldolase A expression levels were significantly higher in cancer compared to non-cancer regions. In vitro studies showed that secretion of aldolase A into the culture medium was clearly suppressed in CRC cancer cells to normal colon epithelium. Finally, aldolase A may play an important role in the carcinogenesis process of CRC and may be useful as an early detection biomarker for CRC.
